# Automated Survey Collection with LLM-based Conversational Agents

**Published:** 2025-04-02

**Authors:** Kurmanbek Kaiyrbekov, Nicholas J Dobbins, Sean D Mooney

**Affiliations:** 1 Cyberinfrastructure and Artificial Intelligence Platforms Section, Center for Genomics and Data Science Research, National Human Genome Research Institute, National Institutes of Health, Bethesda, Maryland, USA; 2 Biomedical Informatics & Data Science, Department of Medicine, Johns Hopkins University, Baltimore, Maryland, USA

**Keywords:** Large language models, surveys, natural language processing, machine learning

## Abstract

**Objective:**

Traditional phone-based surveys are among the most accessible and widely used methods to collect biomedical and healthcare data, however, they are often costly, labor intensive, and difficult to scale effectively. To overcome these limitations, we propose an end-to-end survey collection framework driven by conversational Large Language Models (LLMs).

**Materials and Methods:**

Our framework consists of a researcher responsible for designing the survey and recruiting participants, a conversational phone agent powered by an LLM that calls participants and administers the survey, a second LLM (GPT-4o) that analyzes the conversation transcripts generated during the surveys, and a database for storing and organizing the results. To test our framework, we recruited 8 participants consisting of 5 native and 3 non-native english speakers and administered 40 surveys. We evaluated the correctness of LLM-generated conversation transcripts, accuracy of survey responses inferred by GPT- 4o and overall participant experience.

**Results:**

Survey responses were successfully extracted by GPT-4o from conversation transcripts with an average accuracy of 98% despite transcripts exhibiting an average per-line word error rate of 7.7%. While participants noted occasional errors made by the conversational LLM agent, they reported that the agent effectively conveyed the purpose of the survey, demonstrated good comprehension, and maintained an engaging interaction.

**Conclusions:**

Our study highlights the potential of LLM agents in conducting and analyzing phone surveys for healthcare applications. By reducing the workload on human interviewers and offering a scalable solution, this approach paves the way for real-world, end-to-end AI-powered phone survey collection systems.

## INTRODUCTION

Surveys serve as a vital tool for gathering information and insight on numerous aspects of healthcare, including ensuring an accurate and unbiased assessment of clinical trials [1, 2]. Clinical researchers and practitioners rely on surveys to screen potential participants, monitor their health over time, and ensure adherence to study protocols, making them a cornerstone of evidence-based research [3–5]. Healthcare facilities use surveys to measure patient satisfaction with services, enabling efforts to enhance care quality [6, 7]. Moreover, health surveys empower governments to identify health problems and make informed, data-driven decisions about about development of programs to improve population health. The National Center for Health Statistics (NCHS) conducts continuous surveys to assess the population’s health, track progress toward national health goals [8], estimate health insurance coverage [9], and evaluate care provided in physician offices and health centers [10], among other objectives. These insights provide critical guidance for policymakers.

Surveys can be conducted in various modes that differ in the manner of contacting participants, styling of questions, and the means by which the survey is distributed to participants. Self-administered surveys, such as online and mail-based formats, offer a number of advantages: they provide participants with ample time to respond thoughtfully, minimize interviewer influence, and are often cost-efficient. However, these modalities can pose challenges: mail surveys require participants to visit a post office to return completed forms, and online surveys demand basic digital literacy, which may be a barrier for older individuals. Additionally, these methods are prone to item non-response, where participants skip certain questions [11, 12]. Face-to-face interviews, on the other hand, only require participants to speak the same language as the interviewer. They allow for real-time clarification of questions, verification of responses, and foster personal interaction, which can enhance participant engagement. However, they are resource-intensive, susceptible to interviewer bias, and geographically constrained(e.g., interviewers physically located in one locality cannot easily interview participants 10 hours away).

Phone surveys may offer a viable middle ground. They require only telephone access (which is an increasingly universal resource) and eliminate the need for both interviewer and respondent to be in the same location. Phone surveys combine the benefits of personal engagement through audio communication with the scalability of remote administration. However, they can still be costly, time-intensive for interviewers, and subject to interviewer bias. In this study, we focus on improving the scalability of phone surveys while addressing their limitations by LLMs.

Large language models are foundational AI systems that have demonstrated exceptional performance in natural language processing tasks [13, 14]. Their versatility enables them to be repurposed for applications across various domains, including medicine, where they have showcased impressive reasoning capabilities by excelling in medical exams [15]. LLMs hold significant promise for transforming patient care by analyzing electronic health records and medical image data to generate accurate, interpretable diagnosis [16], supporting clinical decision-making through treatment recommendations aligned with established guidelines [17], determining patients eligible for clinical trials [18], extracting relevant information from clinical notes [19], making health inferences from wearable sensor data [20], concisely summarizing medical evidence [21], and aiding development and discovery of novel drugs [22]. Their robust instruction-following capabilities have driven the emergence of agentic AI systems, where LLMs function as specialized agents to accomplish defined tasks. These systems have been utilized to generate clinical notes from doctor-patient conversations [23], improve performance on medical tests through multi-agent discussions with each agent serving as a specialty expert [24], and produce explainable clinical decisions via collaborative agent interactions [25]. Furthermore, agentic frameworks have been proposed to streamline physicians’ administrative workloads, enhancing operational efficiency and allowing clinicians to dedicate more time to patient care [26, 27].

Multimodal Large Language Models (MMLLMs) have advanced significantly, expanding beyond text processing to handle and synthesize content across various modalities, including images, audio, and video [28]. Among these, speech stands out as the most natural form of human communication. Enabling LLMs to process audio data effectively can make human-AI interactions more intuitive and seamless. Speech-processing LLMs can also be adapted into specialized agents for specific tasks, with one prominent application being telephone agents. These agents are capable of engaging in sophisticated, multilingual conversations, making them particularly well-suited for conducting surveys – especially in the context of healthcare. In this study, we illustrate the potential of AI agents to effectively conduct health-related surveys. We propose a comprehensive AI-driven framework that not only initiates studies and administers surveys but also interprets respondents’ answers and seamlessly uploads the results. We believe this innovative approach could reduce the time and effort required from human personnel, and enhance the accuracy and reliability of large-scale data collection.

## MATERIALS AND METHODS

### Survey Information

We aimed to validate our LLM agent-based framework as effective in conducting real world surveys. To do so, we chose to use an existing survey format, the Covid Impact Survey [29], rather than creating bespoke custom survey questions which may not be directly comparable to rigorously validated published studies. This survey, conducted by the National Opinion Research Center at the University of Chicago, was administered in three waves over several weeks and provided both regional and national statistics about physical health, mental health, economic security, and social dynamics. Its primary goal was to assess the immediate effects of the pandemic on the United States during a time when the federal government was preparing for the 2020 Census and unable to respond quickly. We believe that telephone-based survey collection would have been an ideal method during this period, as it could be implemented at a fast rate at scale by the federal government and aligned well with pandemic precautions such as social distancing.

From the COVID Impact Survey, we selected a subset of 33 questions to include in our study. This number was carefully chosen with comprehensiveness and respondent convenience in mind such that participants could complete one survey in under 10 minutes. While our version of the survey contained many questions related to personal, societal and economic conditions of respondents, the majority of questions were about physical and mental health. All questions required either multiple choice or numeric responses. Detailed information about our adapted survey, including the full list of questions, can be found in the Supplementary Appendix.

### System Overview

**Figure 1:**
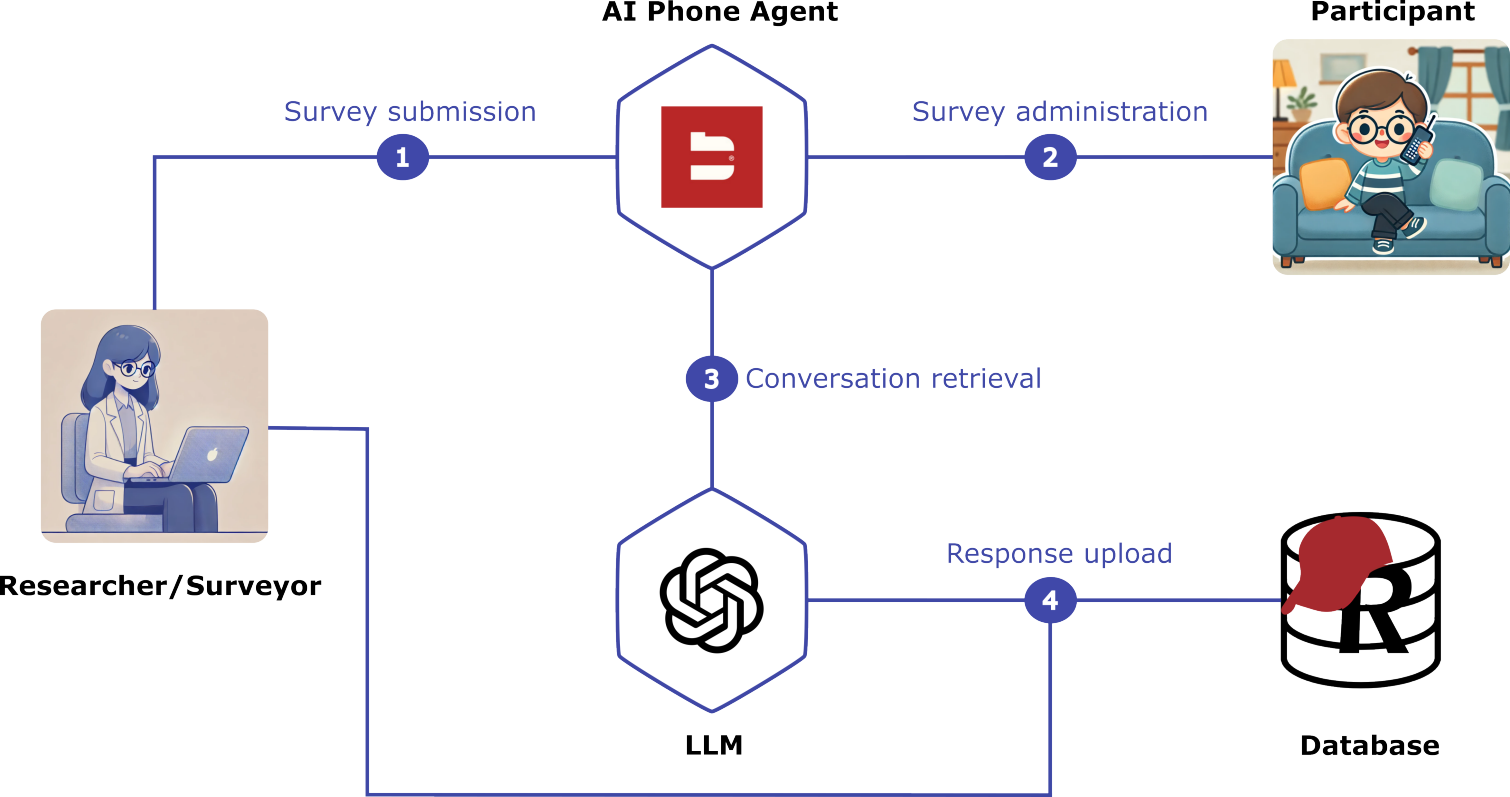
Overview of survey collection and analysis system. Our framework consists of a researcher that prepares a survey and writes necessary prompts for large language models, an AI-based conversable phone agent that calls participants to conducts the survey, a survey participant, a large language model that analyzes conversations to deduce answers to individual survey questions and a database for storing results.

Our system comprises five main components: a researcher, an AI phone agent, the survey participants, a large language model that analyzes the surveys, and a database for storing results (Figure 1). To initiate the process, we prepare the survey and instantiate REDCap – a secure web application designed for building and managing online surveys and databases. Then we collect participants’ phone numbers, and create an instruction prompt for the AI-based phone agent. The instruction prompt is then provided to a conversable LLM-based agent developed by the company BlandAI, which calls participants, conducts the survey, and stores conversation transcripts and audio recordings. We then retrieve the transcripts and prompt GPT-4o to analyze the conversations and deduce answer to each of the questions given by the participant. Finally, we process LLM outputs and upload them into REDCap.

### Study Details

To simulate survey collection and evaluate the quality of data gathered through our framework, we recruited N = 8 participants, primarily members of our research group to serve as survey respondents. The group included 2 female and 6 male participants. Among them, 5 were native English speakers, while 3 spoke English as a second language. This study was reviewed and approved by Institutional Review Board at NIH and granted an exemption, as it involved minimal risk, used publicly available data and did not involve personally identifiable information. Written informed consent was obtained from all participants, who were informed of the voluntary nature of participation and their right withdraw at any point without penalty.

**Figure 2:**
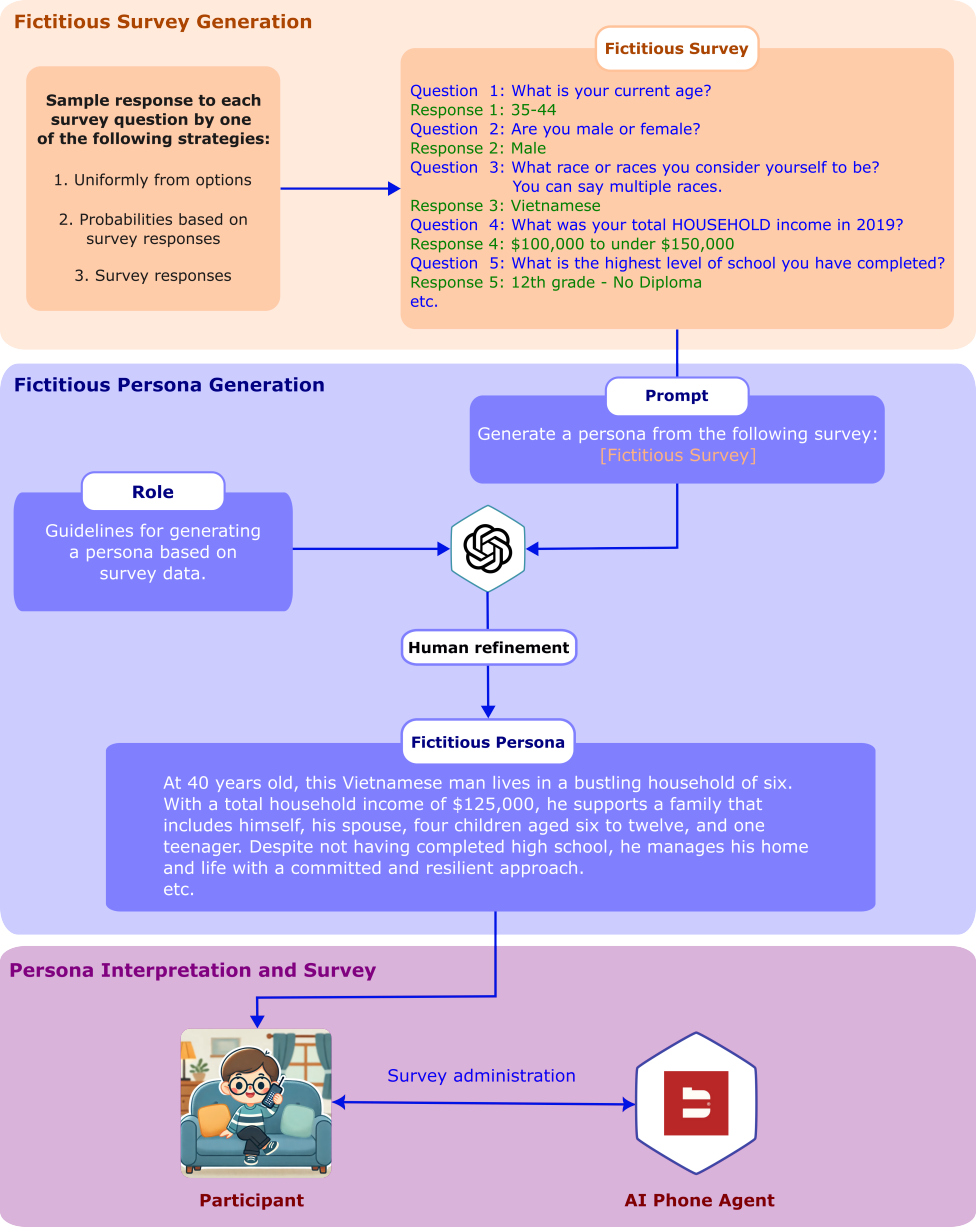
Representation of fictitious persona generation and survey response based on the persona. It consists of 3 main steps: 1) Fictitious survey response generation with answers to each survey question are probabilistically selected from possible options. 2) Fictitious persona generation using GPT-4o based on the fictitious survey. 3) Participant response to survey based on fictitious persona.

Each participant completed the survey five times. Before each survey, they were provided with a description of an imaginary persona containing all the necessary details to answer the survey questions. This approach allowed us to collect multiple responses per participant while avoiding the collection of personally identifiable information. The imaginary personas were generated using GPT-4o [30], the flagship general-use LLM offered by OpenAI at the time of this study and which we determined to be reasonable for various tasks in this study. An overview of persona generation and survey administration process for our study in shown in Figure 2.

To create these personas, we first generated a fictitious conversation by probabilistically selecting responses to each survey question. Three methods were used for this selection: (1) uniform sampling from all possible answer choices, (2) sampling based on probabilities derived from real survey response distributions (e.g., frequencies from a COVID impact survey), or (3) random sampling from actual survey responses. Details regarding the exact generation procedure for each question and the rationale behind each choice can be found in the Supplementary Appendix. Once the fictitious conversation was generated, GPT-4o was then prompted to produce a concise persona description from the transcript. To ensure that this description contained all the information needed for a participant to respond to the survey questions in a manner consistent with the generated conversation, we further checked and refined the personas. Participants were given five minutes to review and familiarize themselves with their assigned persona before speaking with the AI phone agent.

### Evaluations

Our evaluation and scoring focused on three key aspects:

1. LLM phone agent’s transcription of conversations with participants from audio to text.
2. GPT-4o’s performance in deduction of survey responses from conversation transcripts.
3. LLM phone agent’s usability for survey collection and participant experience.

#### Audio to Text Transcription

After the surveys were completed, two study team members listened to all 40 audio recordings. These recordings were securely stored on the Bland platform and were accessible only to the study team. Since the conversation transcripts resulting from the audio conversation were used to infer structured survey responses, the transcription accuracy is crucial. To verify accuracy, we created “correct transcriptions” by reviewing each Bland-generated transcript and correcting any mismatches with the source audio.

We then assessed alignment between the Bland-generated and correct (reference) transcriptions by calculating the Word Error Rate (WER) [31] for each participant’s response. WER measures the percentage of mis-transcribed words and is computed as follows:

(1) $$WER=\frac{S+D+I}{N}$$

Where:

- S is the number of substitutions (words incorrectly replaced).
- D is the number of deletions (words that were omitted).
- I is the number of insertions (extra words that were added but should not be there).
- N is the total number of words in the correct transcription (reference).

We used the Python package *jiwer* to calculate WER. Before computing WER, we standardized each response by removing empty strings, punctuation, and extra spaces; converting all text to lowercase; trimming leading and trailing spaces; and expanding common English contractions (e.g., “let’s” to “let us”).

#### Text Transcriptions to Structured Survey Responses

Next, we evaluated how well a reasoning LLM (GPT-4o) could infer participant responses from potentially error-prone transcripts generated by Bland. We began by listening to all survey recordings and determining the correct responses to each question. For each transcript, we then prompted GPT-4o to deduce the survey answers based on the transcript text (as described in the Supplementary Appendix). To increase reliability, we repeated this inference process five times per transcript and adopted a self-consistency prompting approach [32], selecting the most common answer as the final response. The individual responses to survey questions were enforced to be in correct format and data types by the Python package *pydantic*. Lastly, we measured the accuracy of GPT-4o’s chosen answers to gauge the correctness of the survey responses ultimately stored in the REDCap database.

#### Usability of Phone Agent and Participant Experience

Finally, we aimed to understand participants’ perceptions of, and experiences with, AI-based phone agents and to assess the usability of these agents. We developed a post-study questionnaire consisting of ten Likert-scale items and included an open-ended question for free-form feedback about the agent. Our questionnaire was primarily adapted from Chatbot Usability Questionnaire (CUQ) [33] that assessed the personality, intelligence, error handling, and navigation of chatbots. We repurposed the CUQ to assess our LLM phone agent. Further details on the questionnaire are provided in the Supplementary Appendix.

## RESULTS

Resulting WER scores of the generated transcripts are shown in Table 1. The average WER across all participants was 7. 7%, with the mean error rates for non-native group being slightly larger than the error rate for native speakers (9.6% versus 6.4%).

Although the conversation transcripts had transcriptions errors, the reasoning LLM (GPT-4o) was able to infer responses for survey questions with high accuracy as shown in Table 2. The accuracies indicate no definitive correlation between Word Error Rate (WER) and accuracy. For example, participants 3 and 7 exhibited high WER in their transcriptions; however, the resulting accuracy of response deductions varied significantly – accuracy was lower for participant 3 but remained high for participant 7. Furthermore, despite the higher transcription error rates observed among non-native speakers, GPT-4o achieved slightly greater inference accuracy for these respondents compared to native speakers.

**Figure 3:**
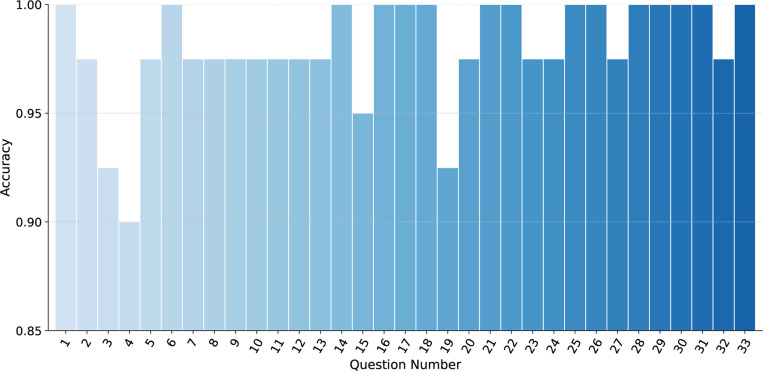
Average accuracy for each survey question. To compute the average accuracy, the accuracy for each question was calculated for each participant as the percentage of correct responses across five personas. The final averages were then obtained by aggregating the accuracies across all participants.

GPT-4o deduced fully correct answers for about half of the questions and word-level accuracy above 97 % for most of the questions (see Figure 3). The lowest score was on question 4 about the total household income. Accuracies are thus reasonably high across participants and for each of the questions.

**Figure 4:**
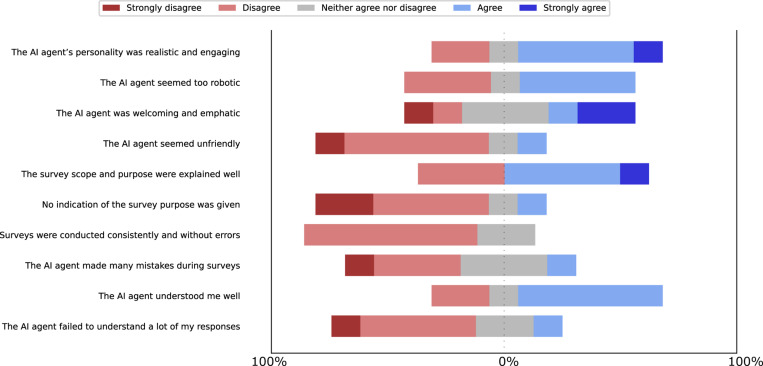
Results from the post-survey questionnaire, illustrating participants’ agreement with various statements. Each statement is listed on the right, accompanied by horizontal bars that represent the proportion of participants selecting each response option. Bar colors correspond to specific response categories, as indicated in the legend.

As indicated by the results of the post-survey questionnaire (Figure 4), the majority of participants found the phone agent to be realistic and engaging, effectively explaining the purpose of the survey and demonstrating a good understanding of their responses during the surveys. Although participants acknowledged that the surveys were not entirely error-free, they did not perceive the AI agent to frequently make mistakes. Opinions were divided on whether the AI agent was empathetic, but most participants (6/8) agreed that the agent was not unfriendly. Interestingly, despite recognizing that the agent seemed realistic, many participants (4/8) also felt that it came across as slightly robotic. Additionally, in response to open ended question “What were the disadvantages of engaging with an AI agent, and what improvements could be made?”, the participants mentioned that it occasionally misunderstood words for instance by confusing “male” with “mail”, changed voice tone, cut off while participant was talking and sometimes spoke too fast among other things (refer to Table S2 for complete reviews). We believe these feedback offer valuable insights to drive future improvements.

## DISCUSSION

We found that using AI agents to conduct and analyze health-related phone surveys was highly effective, demonstrating real-world feasibility for end-to-end AI-powered survey collection. GPT-4o achieved an average response deduction accuracy of 98% from conversation transcripts across eight participants, despite audio transcriptions exhibiting an average word error rate (WER) of 7.7%. We believe these results could be further enhanced by leveraging more advanced large language models, such as OpenAI’s o1, and by reducing transcription errors. While participants acknowledged that the conversational AI agent was not entirely error-free, they generally felt it made few mistakes during the survey. They reported that the agent clearly explained the purpose of the survey, understood the conversation well, and was engaging. However, some felt the agent sounded slightly robotic – a limitation we expect to improve with future iterations that focus on making the agent more human-like. Overall, while the current framework is already highly effective, continued advancements could make AI-driven survey collection virtually indistinguishable from interactions with human agents.

We envision our framework as a complementary tool to existing survey administration methods in biomedical research, such as computer-based questionnaires and face-to-face interviews, with each being used when most appropriate. By acting as an objective surveyor, the AI agent has the potential to reduce interviewer bias, a known issue where interviewer perceived expectations can influence participants to provide conforming responses. Given that current large language models demonstrate fluency in many languages, we believe this framework could also be effective for conducting surveys with international participants. In the current study, we demonstrate existing models and our framework are able to conduct surveys and gather responses reasonably well for both native- and non-native-English speakers of various backgrounds. We plan to expand this line of research by using multilingual AI agents in future.

Another strength of this study is that our methods demonstrate notable potential for scaling and efficiency at less cost – across significantly more participants of greater diversity – than traditional methods. Many biomedical studies and clinical trials are challenged by the need to recruit and maintain participants, often suffering from low statistical power. Moreover, findings of traditional study methods are also challenged by poor potential for generalizability due to participants’ demographics being unrepresentative of larger target populations (e.g., participants living in closer physical proximity to a medical center may be more likely to be recruited, but also more affluent and less diverse than the general population). While AI-driven studies are not a panacea for these challenges, we believe they hold great potential in vastly widening potential participation opportunities for the general population as well as simplifying operations and reducing costs for research teams. Indeed, the total costs to conduct our experiments was $30.15 (BlandAI: $23.92, OpenAI: $6.23), averaging $0.75 per survey. In future real-world scenarios, we envision that such surveys could be automatically administered to hundreds, thousands, or even millions of participants in parallel.

Additionally, large standardized instrument databases such as Phen-X, which utilize common data elements (CDEs), offer an excellent foundation for integration. We believe many of these instruments can be seamlessly adapted for telephone-based survey administration and we intend to incorporate this capability into our framework. While our current focus is on biomedical research surveys, we see broad potential for this framework across other domains, including government census efforts, industry-specific surveys, and a wide range of academic research studies.

### Limitations

This project has some limitations. To avoid collecting personal information and to allow multiple surveys from a single participant, we asked participants to respond based on fictitious personas generated by GPT-4o and refined by our team. However, we observed that this approach led to interactions with the AI agent feeling less natural than if participants had been sharing their own experiences. Although we emphasized that it was not necessary to strictly adhere to the persona, and allowed participants to deviate if unsure, they occasionally paused during the call to search for information in the persona description. This behavior is atypical in natural conversations and may have impacted the flow of the interaction. Additionally, the fact that participants were aware they were speaking with an AI agent may have influenced their behavior. For example, a few participants noted that they wanted to revise an answer after responding, but refrained from doing so because the agent had already moved on to the next question. They assumed it was not possible to go back. We believe that if participants had thought they were speaking with a human, they would have felt more comfortable requesting to revise their responses.

We noticed that the phone agent sometimes wouldn’t enforce the answer format, which in some cases led to ambiguous responses. Also, for some of the questions, such as questions asking about race and gender, we did not instruct the agent to list all of the response options during the survey because they had 10+ options, and we believed that the options were exhaustive. However, some of the responses were ambiguous. For example, for the question about the highest education level completed, one of the participants said, “didn’t finish high school”, which was ambiguous since he/she could have left school at 10th, 11th, or 12th grade, which were all separate response options. In such cases, we labeled the answer as “OTHER” and wrote in the instruction prompt to GPT-4o to also choose “OTHER” for such cases, but there were instances where GPT-4o did not choose the correct label. Similarly, another issue arose when participants gave an answer that could be construed to match multiple response options. In these cases, we felt it reasonable to choose the last uttered option as the correct response and instructed GPT-4o to do so as well. However, there were cases where GPT-4o made mistakes in this scenario. For example, for the question on whether the state of health is good, excellent, fair, or poor, the response in one of the surveys was “Good. Fair.” We labeled the answer as “Fair”, but GPT-4o chose “Good” as the answer. Finally, in the question about whether a person worked for pay at a job or business, even though the AI agent provided options asking if the surveyee worked for someone, was self-employed, or did not work, some of the respondents simply, “Yes.” In this case, we labeled such responses as “Yes, I worked for someone.” GPT-4o did not err in such scenarios. Even though such cases were very rare, we believe that the study design could have been improved if we had predicted them before the study, and potentially GPT-4o would have shown better performance.

## CONCLUSION

This study introduces an LLM-based framework for conducting phone surveys in healthcare and biomedical research. To illustrate the effectiveness of our framework, we conducted a study where we collected 40 surveys from 8 study participants using an AI conversational phone agent and analyzed the resulting surveys with GPT-4o. Our framework demonstrated 98% accuracy in identifying survey responses correctly. In the future, we plan to add automatic adaptation of available survey instruments in Phen-X, and we envision that the performance of our framework will improve with better LLMs.

## Supplementary Material

Supplement 1

## Figures and Tables

**Table 1:** Per-line Word Error Rate (WER) scores for generated transcripts, categorized by speaker groups and aggregated by personas for each participant.

Group	Native Speakers					Non-Native Speakers		
**Participant**	1	2	3	4	5	6	7	8
**WER**	5.1	6.7	10.6	4.9	5.0	6.0	19.7	3.2
**Average WER**	6.4					9.6		

**Table 2:** Average accuracy scores by speaker groups, calculated across all survey questions and aggregated by personas for each participant. All surveys were conducted in english.

Group	Native Speakers					Non-Native Speakers		
**Participant**	1	2	3	4	5	6	7	8
**Accuracy**	98.8	96.4	95.8	99.4	98.2	99.4	97.6	98.8
**Average Accuracy**	97.7					98.6		
