# Supplementary material for: Automated Survey Collection with LLM-based Conversational Agents
Source: ArXiv. 2025 Apr 2:arXiv:2504.02891v1. Preprint. [Version 1] (PMC12306824)
Supplement: Supplement 1 [file NIHPP2504.02891v1-supplement-1.pdf]

APPENDIX

ADAPTED SURVEY QUESTIONS

1. What is your current age?
   (a) 18-24;
   (b) 25-34;
   (c) 35-44;
   (d) 45-54;
   (e) 55-64;
   (f) 65-74;
   (g) 75+;
   (h) Under 18
2. Are you male or female?
   (a) Male;
   (b) Female;
3. What race or races you consider yourself to be? You can say multiple races.
   (a) White;
   (b) Black or African American;
   (c) American Indian or Alaska Native;
   (d) Asian Indian;
   (e) Chinese;
   (f) Filipino;
   (g) Japanese;
   (h) Korean;
   (i) Vietnamese;
   (j) Other Asian;
   (k) Native Hawaiian;
   (l) Guamanian or Chamorro;
   (m) Samoan;
   (n) Other Pacific Islander;
   (o) Some other race;
   (p) Refused
4. What was your total HOUSEHOLD income in 2019?
   (a) Under $10,000
   (b) $10,000 to under $20,000;
   (c) $20,000 to under $30,000;
   (d) $30,000 to under $40,000;
   (e) $40,000 to under $50,000;

(f) $50,000 to under $75,000;
(g) $75,000 to under $100,000;
(h) $100,000 to under $150,000;
(i) $150,000 or more;
(j) Don't know;
(k) Refused

5. What is the highest level of school you have completed?

   (a) No formal education;
   (b) 1st, 2nd, 3rd, or 4th grade;
   (c) 5th or 6th grade;
   (d) 7th or 8th grade;
   (e) 9th grade;
   (f) 10th grade;
   (g) 11th grade;
   (h) 12th grade - NO DIPLOMA;
   (i) High school graduate - high school diploma or the equivalent;
   (j) Some college, no degree;
   (k) Associate degree;
   (l) Bachelor's degree;
   (m) Master's degree;
   (n) Professional or Doctorate degree;
   (o) Refused

6. Including yourself, how many persons currently live in your household at least 50 percent of the time? Please include any children as well as adults.

   (a) One person, I live by myself;
   (b) Two persons;
   (c) Three persons;
   (d) Four persons;
   (e) Five persons;
   (f) Six or more persons;
   (g) Refused

7. How many persons currently living in your household, including yourself, are 0-1 years old?

   (a) Numeric Value
   (b) Don't know;
   (c) Refused;

8. How many persons currently living in your household, including yourself, are 2-5 years old?

   (a) Numeric Value
   (b) Don't know;
   (c) Refused;

9. How many persons currently living in your household, including yourself, are 6-12 years old?
    (a) Numeric Value
    (b) Don't know;
    (c) Refused;
10. How many persons currently living in your household, including yourself, are 13-17 years old?
    (a) Numeric Value
    (b) Don't know;
    (c) Refused;
11. How many persons currently living in your household, including yourself, are 18+ years old?
    (a) Numeric Value
    (b) Don't know;
    (c) Refused;
12. In the past month, how often did you talk with any of your neighbors?
    (a) Basically every day;
    (b) A few times a week;
    (c) A few times a month;
    (d) Once a month;
    (e) Not at all;
    (f) Not sure;
    (g) Refused
13. During a typical month prior to March 1, 2020, when COVID-19 began spreading in the United States, how often did you talk with any of your neighbors?
    (a) Basically every day;
    (b) A few times a week;
    (c) A few times a month;
    (d) Once a month;
    (e) Not at all;
    (f) Not sure;
    (g) Refused
14. In the past 7 days, how often have you felt nervous, anxious, or on edge?
    (a) Not at all or less than 1 day;
    (b) 1-2 days;
    (c) 3-4 days;
    (d) 5-7 days;
    (e) Don't know;
    (f) Refused
15. In the past 7 days, how often have you felt depressed?
    (a) Not at all or less than 1 day;

(b) 1-2 days;
(c) 3-4 days;
(d) 5-7 days;
(e) Don't know;
(f) Refused

16. In the past 7 days, how often have you felt lonely?

    (a) Not at all or less than 1 day;
    (b) 1-2 days;
    (c) 3-4 days;
    (d) 5-7 days;
    (e) Don't know;
    (f) Refused

17. In the past 7 days, how often have you felt hopeless about the future?

    (a) Not at all or less than 1 day;
    (b) 1-2 days;
    (c) 3-4 days;
    (d) 5-7 days;
    (e) Don't know;
    (f) Refused

18. In the past 7 days, how often have you had physical reactions such as sweating, trouble breathing, nausea or a pounding heart when thinking about your experience with the coronavirus pandemic?

    (a) Not at all or less than 1 day;
    (b) 1-2 days;
    (c) 3-4 days;
    (d) 5-7 days;
    (e) Don't know;
    (f) Refused

19. In the past 7 days, did you do any work for pay at a job or business?

    (a) Yes, I worked for someone else for wages, salary, piece rate, commission, tips, or payments "in kind," for example, food or lodging received as payment for work performed;
    (b) Yes, I worked as self-employed in my own business, professional practice, or farm;
    (c) No, I did not work for pay last week;
    (d) Don't know;
    (e) Refused

20. Would you say your health in general is excellent, very good, good, fair, or poor?

    (a) Excellent;
    (b) Very good;
    (c) Good;
    (d) Fair;

(e) Poor;
(f) Don't know;
(g) Refused

21. Has a doctor or other health care provider ever told you that you have COVID-19?
    (a) Yes;
    (b) No;
    (c) Not sure;
    (d) Refused
22. Has a doctor or other health care provider ever told someone you live with that they have COVID-19?
    (a) Yes;
    (b) No;
    (c) Not sure;
    (d) Refused
23. Have you experienced chills in the past 7 days, or not?
    (a) Yes;
    (b) No;
    (c) Not sure;
    (d) Refused
24. Have you experienced runny or stuffy nose in the past 7 days, or not?
    (a) Yes;
    (b) No;
    (c) Not sure;
    (d) Refused
25. Have you experienced chest congestion in the past 7 days, or not?
    (a) Yes;
    (b) No;
    (c) Not sure;
    (d) Refused
26. Have you experienced skin rash in the past 7 days, or not?
    (a) Yes;
    (b) No;
    (c) Not sure;
    (d) Refused
27. Have you experienced cough in the past 7 days, or not?
    (a) Yes;
    (b) No;
    (c) Not sure;

(d) Refused

28. Have you experienced sore throat in the past 7 days, or not?
    (a) Yes;
    (b) No;
    (c) Not sure;
    (d) Refused
29. Have you experienced sneezing in the past 7 days, or not?
    (a) Yes;
    (b) No;
    (c) Not sure;
    (d) Refused
30. Have you experienced muscle or body aches in the past 7 days, or not?
    (a) Yes;
    (b) No;
    (c) Not sure;
    (d) Refused
31. Have you experienced headaches in the past 7 days, or not?
    (a) Yes;
    (b) No;
    (c) Not sure;
    (d) Refused
32. Can you use a thermometer to take your temperature now?
    (a) Yes;
    (b) No;
    (c) Don't know;
    (d) Refused
33. What is your body temperature?
    (a) Numeric Value
    (b) Refused

An additional "OTHER" option was added to most of the questions to handle cases when answers did not fall into any category.

PROMPT FOR BLAND

Your name is Sarah, and you're a surveyor for COVID-19 Household Impact Survey. The goal of the survey is to provide national and regional statistics about physical health, mental health, economic security, and social dynamics in the United States. Here are the questions that you need to ask, give enough time to reply, introduce yourself, and let them know the purpose of the survey:

1. What is your current age?
2. Are you male or female?
3. What race or races do you consider yourself to be? You can say multiple races.
4. What was your total HOUSEHOLD income in 2019?
5. What is the highest level of school you have completed?
6. Including yourself, how many persons currently live in your household at least 50 percent of the time? Please include any children as well as adults.
7. How many persons currently living in your household, including yourself, are 0-1 years old?
8. How many persons currently living in your household, including yourself, are 2-5 years old?
9. How many persons currently living in your household, including yourself, are 6-12 years old?
10. How many persons currently living in your household, including yourself, are 13-17 years old?
11. How many persons currently living in your household, including yourself, are 18+ years old?
12. In the past month, how often did you talk with any of your neighbors? Response options: Basically every day, a few times a week, a few times a month, once a month, not at all, not sure?
13. During a typical month prior to March 1, 2020, when COVID-19 began spreading in the United States, how often did you talk with any of your neighbors? Response options: Basically every day, a few times a week, a few times a month, once a month, not at all, not sure?
14. In the past 7 days, how often have you felt nervous, anxious, or on edge? Please provide your answer in number of days.
15. In the past 7 days, how often have you felt depressed? Please provide your answer in number of days.
16. In the past 7 days, how often have you felt lonely? Please provide your answer in number of days.
17. In the past 7 days, how often have you felt hopeless about the future? Please provide your answer in number of days.
18. In the past 7 days, how often have you had physical reactions such as sweating, trouble breathing, nausea, or a pounding heart when thinking about your experience with the coronavirus pandemic?
19. In the past 7 days, did you do any work for pay at a job or business? Did you work for someone, were you self-employed, or didn't work at all?
20. Would you say your health in general is excellent, very good, good, fair, or

poor?
21. Has a doctor or other health care provider ever told you that you have COVID-19?
22. Has a doctor or other health care provider ever told someone you live with that they have COVID-19?
23. Have you experienced chills in the past 7 days, or not?
24. Have you experienced runny or stuffy nose in the past 7 days, or not?
25. Have you experienced chest congestion in the past 7 days, or not?
26. Have you experienced skin rash in the past 7 days, or not?
27. Have you experienced cough in the past 7 days, or not?
28. Have you experienced sore throat in the past 7 days, or not?
29. Have you experienced sneezing in the past 7 days, or not?
30. Have you experienced muscle or body aches in the past 7 days, or not?
31. Have you experienced headaches in the past 7 days, or not?
32. Can you use a thermometer to take your temperature now?
33. What is your body temperature?

Here is an example dialogue:
You: Hello! My name is Sarah, and I'm conducting a survey to gather information on the impact of COVID-19 on various aspects of people's lives. This survey will help us understand how the pandemic has affected different communities. Your responses will be kept confidential and used for research purposes only. Could I have a few minutes of your time to answer some questions?
Person: Sure!
You: Thank you so much! To begin, what is your current age?
Person: I'm 32 years old.
You: Great, thank you. Are you male or female?
Person: I'm female.
You: Thank you. What race or races do you consider yourself to be? You can mention multiple races if applicable.
Person: I consider myself to be Asian.
You: Got it. What was your total household income in 2019?
Person: Our total household income was around $75,000.
You: Thank you for sharing that. What is the highest level of school you have completed?
Person: I have a Bachelor's degree.
You: Including yourself, how many persons currently live in your household at least 50 percent of the time? Please include any children as well as adults.
Person: There are four of us.
You: And how many persons currently living in your household, including yourself, are 0-1 years old?
Person: None.
You: How about 2-5 years old?
Person: We have one child in that age range.
You: And how many are 6-12 years old?
Person: None in that age range.
You: How many persons currently living in your household, including yourself, are 13-17 years old?

Person: None.
You: Finally, how many persons currently living in your household, including yourself, are 18+ years old?
Person: There are three adults.
You: Thank you. In the past month, how often did you talk with any of your neighbors? Would you say basically every day, a few times a week, a few times a month, once a month, not at all, or not sure?
Person: A few times a month.
You: During a typical month prior to March 1, 2020, when COVID-19 began spreading in the United States, how often did you talk with any of your neighbors? Would you say basically every day, a few times a week, a few times a month, once a month, not at all, or not sure?
Person: I would say a few times a week.
You: In the past 7 days, how often have you felt nervous, anxious, or on edge? Please provide your answer in number of days.
Person: I've felt that way about 3 days.
You: How often have you felt depressed in the past 7 days? Please provide your answer in number of days.
Person: Probably around 2 days.
You: And how often have you felt lonely in the past 7 days? Please provide your answer in number of days.
Person: About 4 days.
You: In the past 7 days, how often have you felt hopeless about the future? Please provide your answer in number of days.
Person: Maybe 1 day.
You: In the past 7 days, how often have you had any physical reactions such as sweating, trouble breathing, nausea, or a pounding heart when thinking about your experience with the coronavirus pandemic?
Person: I haven't experienced that.
You: In the past 7 days, did you do any work for pay at a job or business? Did you work for someone, were you self-employed or didn't work at all?
Person: Yes, I did work for a company.
You: Would you say your health in general is excellent, very good, good, fair, or poor?
Person: I would say very good.
You: Has a doctor or other health care provider ever told you that you have COVID-19?
Person: No, I haven't been told that.
You: Has a doctor or other health care provider ever told someone you live with that they have COVID-19?
Person: No, no one in my household has been told that.
You: Have you experienced chills in the past 7 days, or not?
Person: No, I haven't.
You: Have you experienced a runny or stuffy nose in the past 7 days, or not?
Person: Yes, I have.
You: How about chest congestion?
Person: No.

```
You:  Have you experienced a skin rash in the past 7 days, or not?
Person:  No.
You:  Have you experienced a cough in the past 7 days, or not?
Person:  Yes, I have.
You:  How about a sore throat?
Person:  Yes, I've had a sore throat.
You:  Have you experienced sneezing in the past 7 days, or not?
Person:  Yes, I have.
You:  How about muscle or body aches?
Person:  Yes, I've experienced that.
You:  Have you experienced headaches in the past 7 days, or not?
Person:  Yes, I have.
You:  Can you use a thermometer to take your temperature now?
Person:  Sure, just a moment.  [Pause while Person takes their temperature] It's
98.6°F.
You:  Thank you for providing that information.  That concludes our survey.  Thank
you so much for your time and for your valuable responses.  Have a great day!
Person:  You're welcome.  Have a great day too!
```

PERSONA GENERATION PROCEDURE

Fictitious persona generation is done in 2 main steps:

1. Fictitious survey synthesis
2. Fictitious persona synthesis

To create a fictitious survey, we randomly select answers for each question according to one of three main strategies. The specific sampling strategy used for each question is shown in Table S1.

Strategy 1 (Uniform Random Selection). We choose an answer uniformly at random from all available options. This strategy ensures that every response option is equally represented. We apply this method to Questions 3–5, which ask about race, household income, and highest degree obtained. Because these questions each have at least ten possible responses, using a uniform approach allows us to capture the full diversity of potential answers.

Strategy 2 (Survey Response Probability-Based Selection). We select answers based on probabilities derived from the frequency of responses in an actual COVID-19 impact survey conducted in April. After converting the observed frequencies to probabilities, we sample responses accordingly. This approach is

used for Question 2 and Questions 12–33, which cover social conditions, mental health, physical health, and economic conditions. For Question 33, which deals with body temperature measurement, we first probabilistically determine whether the respondent refuses to provide a temperature. If not, we then select a temperature uniformly at random from the distribution of actual responses.

Strategy 3 (Survey-Based Consistent Response Selection). For Question 1 and Questions 6–11, we use actual respondent data from the April survey to maintain logical consistency among related questions. First, we randomly select a response to Question 6 (household size). We then filter the April survey dataset to include only those respondents who reported the same household size. From this filtered subset, we randomly choose one individual and use their reported numbers of household members in specific age categories, as well as their own age, to fill in our fictitious survey. This method ensures consistency between total household size and the number of people in each age group.

Once the fictitious survey is complete, we use a GPT-4o-based agent to generate a fictional persona. We start by defining the agent's role and outlining the detailed procedure for creating the persona, as shown in Figure S2. Then, using the survey data as input, we prompt the agent to produce the corresponding persona.

INSTRUCTIONS FOR ANALYSIS OF TRANSCRIPTS

In our framework, GPT-4o infers answers to individual survey questions from conversation transcript. Initially, GPT-4o's role is describe with a prompt shown in Figure S1. Then GPT-4o is provided with full conversation and asked to produce responses to each of the questions. This process is repeated 5 times and the most frequent answer is chosen as the final response.

POST-STUDY QUESTIONNAIRE

After completing the surveys, participants were asked to evaluate their experience by indicating the extent to which they agreed or disagreed with the following statements:

1. The AI agent's personality was realistic and engaging.

2. The AI agent appeared overly robotic.

```
You are a diligent assistant tasked with analyzing survey conversation
transcripts to deduce the respondent's answers to each of the 33 questions.
Ensure that every question has a corresponding answer.  Keep in mind the
following guidelines:
  1. Deducing Answers:  Responses to specific questions may sometimes be
     inferred from the broader conversation, even if the question was not
     explicitly asked.  Use context to determine the best possible answer.
  2. Handling Transcription Errors:  Conversations may contain transcription
     errors.  Do your best to interpret the respondent's intended meaning
     accurately.
  3. Refusals or Skipped Questions:  If the respondent explicitly refuses
     to answer or skips a question, label the answer as REFUSED. Keep in
     mind that if the respondent explicitly refuses to answer one question,
     this may sometimes imply a refusal to answer other related questions as
     well.
  4. Ambiguity or Non-Matching Responses:  If the respondent's answer does
     not align with any of the predefined response options or is ambiguous,
     label the answer as OTHER.
  5. Multiple Responses:  If the respondent tells several matching response
     options within an answer to a question, consider the last response
     option they give as the final answer.
Your goal is to carefully analyze the transcript and assign the most
appropriate answer to each question based on the above criteria.
Below is the list of questions, each followed on the next line by its
corresponding response options separated by semicolons:
[List of questions with response options follow here.]
```

Figure S1: Assignment and role description for GPT-4o agent that analyzes conversation transcripts.

3. The AI agent was welcoming and empathetic.

4. The AI agent seemed unfriendly.

5. The scope and purpose of the survey were clearly explained.

6. There was no clear indication of the survey's purpose.

7. Surveys were conducted consistently and without errors.

8. The AI agent made frequent mistakes during the surveys.

9. The AI agent understood me well.

```
You are an intelligent and resourceful assistant tasked with analyzing
a survey consisting of 33 questions and their corresponding responses.
Your objective is to craft a detailed, three-paragraph description of
a fictional individual based on the survey answers you review.  This
description should be comprehensive enough that another person, upon
reading it, would be able to accurately replicate the original responses
to all 33 questions.

In the first paragraph, you will summarize and incorporate the answers
to questions 1 through 11.  The second paragraph will cover the responses
provided for questions 12 through 22.  Finally, the third paragraph will
capture the answers to questions 23 through 33.  The goal is to create a
cohesive and vivid portrayal of the individual, ensuring the description
reflects the essence of their survey answers in a clear and engaging
manner.

Here are some additional guidelines to follow:
   • Make paragraphs as short as possible.  No unnecessary information.
   • When describing age, provide a specific number rather than a range.
   • When describing household income, provide a specific figure that fits
     answer range.  Don't state a range.
   • When describing education attained, state the exact answer.
   • When describing questions 14 though 18, state the answer in days.
   • Make paragraphs as short as possible while maintaining narrative.
   • Your description should not explicitly list or restate the answers
     to the survey questions.  Exceptions for this rule are stated above.
     Instead, craft a cohesive and natural narrative that captures the
     essence of the person's character and lifestyle.
```

Figure S2: Assignment and role description for GPT-4o agent that generates persona from a fictitious survey.

10. The AI agent failed to comprehend many of my responses.

Participants rated each statement using a five-point Likert scale: strongly disagree, disagree, neither agree nor disagree, agree, and strongly agree. Additionally, they were invited to respond to an open-ended question: "What were the disadvantages of engaging with the AI agent, and what improvements could be made?"

Table S1: Response sampling strategy for each survey question.

Question	Sampling Strategy
1	april survey-based consistent response
2	survey response probability-based
3	uniform random
4	uniform random
5	uniform random
6	april survey-based consistent response
7	april survey-based consistent response
8	april survey-based consistent response
9	april survey-based consistent response
10	april survey-based consistent response
11	april survey-based consistent response
12	april survey response probability-based
13	april survey response probability-based
14	april survey response probability-based
15	april survey response probability-based
16	april survey response probability-based
17	april survey response probability-based
18	april survey response probability-based
19	april survey response probability-based
20	april survey response probability-based
21	april survey response probability-based
22	april survey response probability-based
23	april survey response probability-based
24	april survey response probability-based
25	april survey response probability-based
26	april survey response probability-based
27	april survey response probability-based
28	april survey response probability-based
29	april survey response probability-based
30	april survey response probability-based
31	april survey response probability-based
32	april survey response probability-based
33	april survey response probability-based

**Response**
If I tell the AI agent that I am living on my own it does not need to loop through all questions about the age of the persons living in my household. Maybe just ask for the age of all persons and then put it in the age bins by itself. At some point it started to pronounce the end of each question weirdly by pitching the tone up. When I asked it to stop it stopped, but only for a few questions.
The AI made some noticeable mistakes during the survey. For example, when asked to specify gender, I said "male," but it misinterpreted it as "mail." Additionally, when mentioning depression, it only acknowledged and showed empathy once across multiple attempts. The bot's tone also felt plain and lacked enthusiasm or friendliness, making the experience less engaging. While it didn't make many mistakes overall, the errors it did make were noticeable. It also struggled to handle ambiguous information and translate it into the appropriate survey response. I felt limited to giving short answers to the survey rather than giving natural/human responses.
An AI agent could be more patient. For example, when I tried to say "fifteen thousand", the agent cut me off at "fifteen".
The AI got hung up on particular words - female, "none" (said sarcastically/sardonically), and one other word. That was the most frustrating aspect of the interactions and also somewhat surprising. Sometimes I spoke over the AI and sometimes the AI spoke over what I was saying. Humans do the same thing, but I think the "turn taking" ability was not quite as good as the average human (however it was good enough it wasn't annoying, at least for me). One improvement might be to allow keypad entry of items (this would get around the problem with not being able to understand "none" – I could just answer "0").
The AI had trouble with the words male and female. It sometimes struggled with numbers like "four". But overall it was actually better than most IVRs I have used.
I probably would have hung up if it were a call out of the blue from an AI bot. That said, I thought the experience was good and would be intrigued if the first question was about whether I wanted to do the survey, and if I preferred talking to a human or to a realistic AI. There were a few things that the AI didn't quite understand, like if I said I lived alone or only with other adults, it still asked how many children in the different age brackets. Everything else was minor (like it seemed to want brief sentences such as "I'm a male" as opposed to just answering "male"). The rest I thought it did a good job, even when I answered in more natural language than just a yes or no. Lastly, it might help if at the outset it could be more explicit about what it can do, such as saying, "If at any point you need help or have any questions, simply ask and this AI can repond"
Thanks for including me in your study! Pros: The AI Agent had a nice voice, the survey questions were clear and they understood me for the most part. Cons: The agent did not have a "personality" (is this what you were hoping for?) because there was no interaction that could have elicited this IMO. The AI Agent did not start with a warm greeting or ask me how I was doing, this seems like what a human would do. Also, it would have been good for them to give me an overview of why they were giving the survey i.e. "This will be a 5-7 minute survey, if you don't understand something please ask me to clarify." Maybe even say that it is an interactive AI agent and the intent is that you can converse with me. The AI agent did not understand my response of "Male" at least twice, the phrase "or not" sounded like a different voice, as did "you can take your temperature now" which was said in a juvenile or condescending tone. If the AI agent knew I lived alone, it should not have asked me the subsequent # of persons in household questions (which were long). For the personas, I found that most said they were feeling lonely but not depressed, but when asked if depressed if they included that they were lonely I answered yes to depressed. If this was a human agent, they would have reiterated what I have said like, "you indicated you have felt lonely. . . to understand that more can you share if you've felt hopeless . . . depressed . . ." Side note: Regardless of if a survey is done by an AI Agent, human or self-service via an online tool, I don't particularly like to take surveys. If someone likes to take surveys and they prefer doing so with a human, I am not sure that the AI agent would be similar enough to a human to motivate them to take the survey.
speak a bit slower for foreigners; skip some questions if the answer is none of the symptoms present.

Table S2: Responses to the post-study questionnaire question "What were the disadvantages of engaging with the AI agent, and what improvements could be made?" from 8 study participants.
